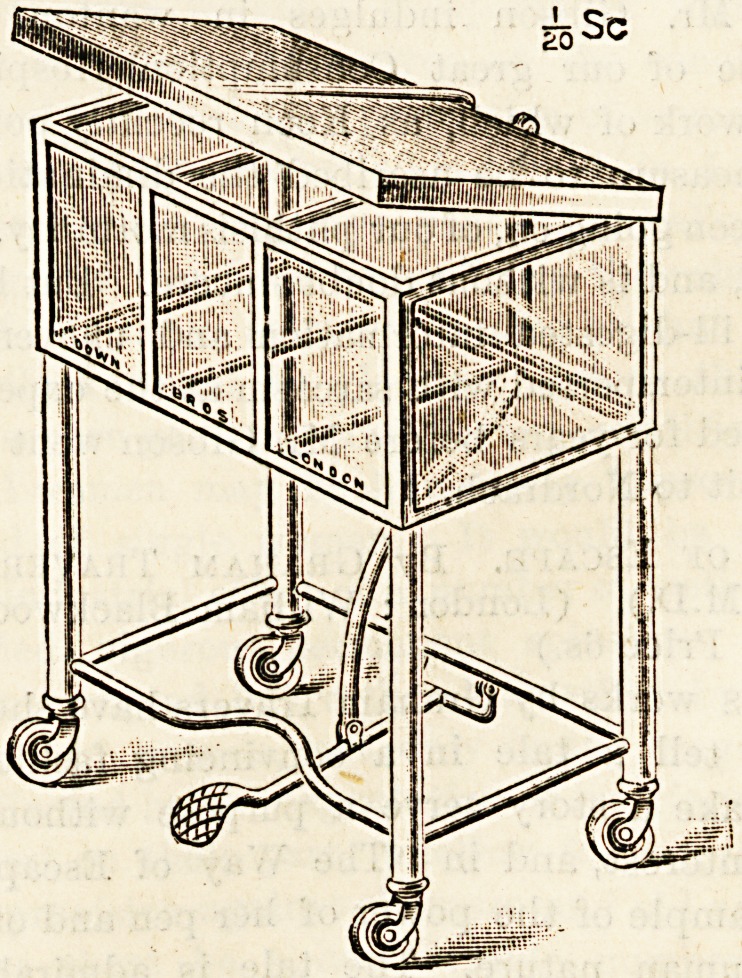# New Appliances and Things Medical

**Published:** 1902-08-02

**Authors:** 


### NEW APPLIANCES AND THINGS MEDICAL.

**[We shall be glad to receive at our Office, 28 & 29 Southampton Street, Strand, London, W.C., from the manufacturers, specimens of all new preparations and appliances which may be brought out from time to time.]**

### FORCE: A NEW FOOD.

(THE FORCE FOOD COMPANY, PENINSULAR HOUSE, MONUMENT STREET, LONDON, E.C.

"FORCE" is a new variety of ready-cooked breakfast dish; it may advantageously be employed as a substitute for oatmeal, and other cereal preparations of this class. Its virtues as a dietetic preparation lie in the possession of the following properties: it is completely cooked and ready for use; it consists of whole wheat, subjected to a high temperature and combined with barley malt. "Force" is therefore partially digested, it is highly nutritious, and it is crisp and deliciously flavoured. It should be an ideal food for growing children and those engaged in occupations which necessitate the expenditure of much physical energy.

### ASEPTIC CABINET.

(MESSRS. DOWN BROS., 21 ST. THOMAS STREET, LONDON, E.C.)

THE accompanying illustration represents a new form of aseptic cabinet for holding surgical instruments or dressings. It is made entirely of glass and metal, and in part it is white enamelled for the sake of neatness and the prevention of rust. The cabinet runs easily and smoothly on castors, which are provided with ball bearings, so that it can be run noiselessly up and down the ward of a hospital or infirmary. The new cabinet possesses the advantage of enabling the nurse to dress a case single-handed with complete aseptic precaution, for after cleansing the hands, the lid can be opened by means of a foot-lever, thus rendering it unnecessary to touch the outside of the cabinet with the hands.

## Figures and Tables

**Figure f1:**